# Poisoning the Family

**Published:** 1874-08

**Authors:** 


### POISONING THE FAMILY.

If a lead pipe, which conveys drinking water into a dwelling, has an indentation, or a too sharp turn, or an orifice hardly large enough to admit a pin, lodgments of particles are made ; these being kept wet, decompose the lead pipe, set the poison free, the family take a little of it day by day, and in weeks, or months, or years, one or more of the members begin to sicken without appreciable cause ; some go abroad and get well, others pine away ; some who are full of vigor, or are away from home a great deal, remain apparently uninjured. In these cases the effects are seen of taking a very little poison into the system every day. A hospitable English family was greatly chagrined from its having been observed in the neighborhood that every visitor to the mansion who remained there several days became ill, but immediately recovered when they returned to their own homes; the family itself had uninterrupted health, servants and all.

The family physician took the matter in hand, and after patient and close investigation, ascertained that the family put their guests into their "spare rooms"—the very best in the building—and that these rooms were the only papered rooms, and that a paper had been used which was covered with green figures, looking like velvet. Some of this "green" was chemically examined, and found to contain dust of arsenic so fine that every breath of air would send it flying into the apartment, and being breathed into the lungs, and swallowed into the stomach with the saliva, it was introduced immediately into the blood, causing arsenical poisoning.

